# Anosmin-1 contributes to brain tumor malignancy through integrin signal pathways

**DOI:** 10.1530/ERC-13-0181

**Published:** 2014-02

**Authors:** Catherine T Choy, Haseong Kim, Ji-Young Lee, David M Williams, David Palethorpe, Greg Fellows, Alan J Wright, Ken Laing, Leslie R Bridges, Franklyn A Howe, Soo-Hyun Kim

**Affiliations:** 1 Division of Biomedical SciencesSt George's Medical School, University of LondonCranmer Terrace, London, SW17 0REUK; 2 Academic Neurosurgery UnitSt George's Medical School, University of LondonCranmer Terrace, London, SW17 0REUK; 3 Division of Clinical SciencesSt George's Medical School, University of LondonCranmer Terrace, London, SW17 0REUK; 4 Department of Cellular PathologySt George's Medical School, University of LondonCranmer Terrace, London, SW17 0REUK; 5 Division of Cardiac and Vascular SciencesSt George's Medical School, University of LondonCranmer Terrace, London, SW17 0REUK; 6 Department of Electrical and Electronic EngineeringImperial College LondonExhibition Road, London, SW7 2AZUK

**Keywords:** anosmin-1, Kallmann syndrome, brain tumor, integrins, matrix metalloproteinases, meta-analysis, tumor microenvironment

## Abstract

Anosmin-1, encoded by the *KAL1* gene, is an extracellular matrix (ECM)-associated protein which plays essential roles in the establishment of olfactory and GNRH neurons during early brain development. Loss-of-function mutations of *KAL1* results in Kallmann syndrome with delayed puberty and anosmia. There is, however, little comprehension of its role in the developed brain. As reactivation of developmental signal pathways often takes part in tumorigenesis, we investigated if anosmin-1-mediated cellular mechanisms associated with brain tumors. Our meta-analysis of gene expression profiles of patients' samples and public microarray datasets indicated that *KAL1* mRNA was significantly upregulated in high-grade primary brain tumors compared with the normal brain and low-grade tumors. The tumor-promoting capacity of anosmin-1 was demonstrated in the glioblastoma cell lines, where anosmin-1 enhanced cell motility and proliferation. Notably, anosmin-1 formed a part of active β1 integrin complex, inducing downstream signaling pathways. ShRNA-mediated knockdown of anosmin-1 attenuated motility and growth of tumor cells and induced apoptosis. Anosmin-1 may also enhance the invasion of tumor cells within the ECM by modulating cell adhesion and activating extracellular proteases. In a mouse xenograft model, anosmin-1-expressing tumors grew faster, indicating the role of anosmin-1 in tumor microenvironment *in vivo*. Combined, these data suggest that anosmin-1 can facilitate tumor cell proliferation, migration, invasion, and survival. Therefore, although the normal function of anosmin-1 is required in the proper development of GNRH neurons, overexpression of anosmin-1 in the developed brain may be an underlying mechanism for some brain tumors.

## Introduction

The extracellular matrix (ECM) is fundamentally involved in brain development, regulating the proliferation and migration of neuronal precursors, axonal guidance, and synapse formation. The ECM also plays a critical role during neoplastic transformation. Anosmin-1 is a secreted ECM-associated protein encoded by the *KAL1* gene. Loss-of-function mutations of *KAL1* underlie Kallmann syndrome (KS), a developmental disorder characterized by the association of hypogonadotrophic hypogonadism and anosmia. KS is caused by the defective migration of the gonadotrophin-releasing hormone (GNRH) neurons along the olfactory axonal pathways during early forebrain development (Schwanzel-Fukuda *et al*. 1989). KS is a form of secondary hypogonadism due to the insufficient hypothalamic secretion of GNRH, resulting in low plasma-luteinizing hormone and follicle-stimulating hormone (Hoffman & Crowley 1982).

We and others have shown that anosmin-1 is a guidance cue (Cariboni *et al*. 2004) and a chemo-attractant for the GNRH neurons (Hu *et al*. 2009). In human embryonic GNRH neuroblasts, anosmin-1 induces neurite outgrowth and cell migration through fibroblast growth factor receptor 1 (FGFR1) pathways (Gonzalez-Martinez *et al*. 2004). Anosmin-1 directly binds to FGFR1 and modulates its signaling (Hu *et al*. 2009), and mutations of FGFR1 and its ligand FGF8 have been found in KS (Falardeau *et al*. 2008). Anosmin-1 also binds to urokinase plasminogen activator (uPA), enhancing its proteolytic activity *in vitro* and induces uPA-dependent cell proliferation (Hu *et al*. 2004). Anosmin-1 contains evolutionarily conserved fibronectin type III (FNIII) domains (Choy & Kim 2010) which may interact with multiple signaling complexes on the cell surface. Both FGF/FGFR and uPA/uPAR complexes interact with integrins (Wei *et al*. 1996, Mori *et al*. 2008). Targeted knockout of β1 integrin impaired GNRH neuronal migration in mouse, resulting in delayed puberty and reproductive dysfunctions (Parkash *et al*. 2012), suggesting an important role of β1 integrin in GNRH ontogeny. However, whether anosmin-1 regulates integrin function is unknown. Integrin plays major roles in tumorigenesis (Maglott *et al*. 2006, Brown *et al*. 2008), mediating anchorage-independent cell growth and survival. Detachment from the ECM causes a programed cell death, termed anoikis.

Although much research has focused on the role of anosmin-1 during development, there is little evidence for the function of anosmin-1 in the developed brain. According to the NCBI AceView Tissue Expression data, anosmin-1 is expressed in various normal and pathologic tissues, including the brain, reproductive systems, and neuroendocrine tumors. Reactivation of developmental programs in the adult often associates with pathological conditions (Thiery *et al*. 2009), and the gene expression profiles of tumors possess a considerable resemblance to embryonic stem cells (Ben-Porath *et al*. 2008). We hypothesized that anosmin-1-mediated signal pathways might contribute to brain tumorigenesis. To systematically address this idea, we examined the *KAL1* gene expression in brain tumor microarray datasets from Gene Expression Omnibus (GEO) and our own patients' samples. These revealed that *KAL1* was differentially expressed according to the grade and type of tumor, showing an upregulation in high-grade primary brain tumors. We also found that anosmin-1 enhanced proliferation and motility of glioblastoma cells *in vitro*, formed a complex with integrin β1 inducing downstream signaling, and modulated cell adhesion. Knockdown of *KAL1* decreased tumor cell motility and proliferation, but increased apoptosis. Moreover, anosmin-1 increased the extracellular protease activities, supporting its role in tumor invasion. Finally, anosmin-1-expressing tumors exhibited more aggressive behavior *in vivo*. These data demonstrate novel functions of anosmin-1 in tumor microenvironment, suggesting a common signal pathway involved in GNRH system and brain tumorigenesis.

## Subjects and methods

### Meta-analysis of public microarray data

We used the rank-product method, which ranks the ratio of expression levels and tests the significance which is a product of the corresponding ranks. Normal (N), low (L), and high (H) groups were analysed in pairs (NL, LH, NH) to identify differentially expressed genes. Suppose the case with NL comparison and let *X*_*i,j*__*,k*_ be the logarithm taken the *i*th probe expression of *j*th sample in the *k*th group (*i*=1,…,*p*,*j*=1,…,*n*,*k*∈*N*,*L*,*H*). Then we can compute the ratio of the *i*th probe between the expressions of the *j*th sample in the low group and the *l*th sample in normal group, *Y*_*i,*__*m*_=*X*_*i,j*__*,N*_–*X*_*i,*__*l,L*_*m*={1,…,*M*(=*n*_*N*_×*n*_*L*_)}, where *n*_*N*_ and *n*_*L*_ are the total number of samples in normal and low groups respectively. After ranking the *Y*_*i,m*_ among *M* comparisons, the rank-product statistic *RP*_*i*_ of the *i*th probe can be computed by 
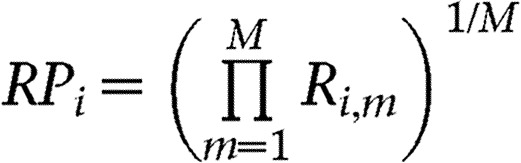
 where *R*_*i,m*_ is the rank of *Y*_*i,m*_. In order to generate the null distribution for our hypothesis, the samples are randomly permuted for each probe. The permuted rank-product statistics *RP*_i_^*^ is computed in the same way as *RP*_*i*_. The empirical *P* values of *RP*_*i*_ can be obtained by


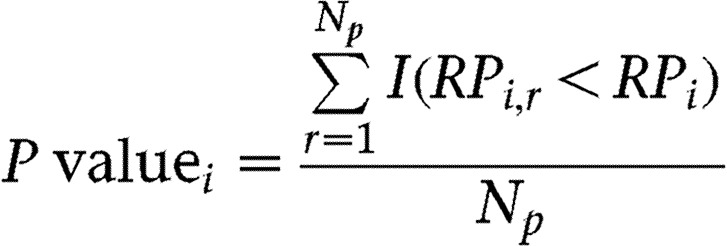


(*N*_*p*_, total permutations which is 5000; *RP*_i_^*^, permuted rank-product statistics; *I*(*C*) is 1 if *C* is true or zero otherwise.) If the *i*th probe is higher in low group than normal group, its rank-product statistic tends to be small with the empirical *P* value close to zero, rejecting the null hypothesis.

### Brain tumor biopsy sample analysis

The tissue samples were collected from consenting patients undergoing surgery for brain tumors at St George's Hospital, as part of the eTUMOUR project http://solaria.uab.es/eTumour/ (Julia-Sape *et al*. 2012). Microarray analysis was performed on Affymetrix HG-U133 Plus 2.0 chips from snap–frozen tissues. For qRT-PCR, RNA was extracted from the formalin-fixed paraffin-embedded (FFPE) tissues using RNeasy FFPE kit (Qiagen). cDNAs were amplified using the Light-Cycler plus SYBR green (Roche) in Light-Cycler 2.0 (Supplementary Table 1, see section on supplementary data given at the end of this article).

### Cell culture

LN229 (CRL-2611), A172 (CRL-1620), and U87MG (HTB-14) were purchased from American Type Culture Collection (ATCC, Manassass, VA, USA) in December 2007. ATCC routinely authenticates these lines by isoenzyme (interspecies) and STR (intraspecies) analysis. All experiments were performed at passages <25–30. Cells were cultured in DMEM, supplemented with antibiotics and 5–10% FBS, at 37 °C with 5% CO_2_. Cells were transfected with Fugene (Roche) and selected using 2 μg/ml puromycin or 400 μg/ml G418 (Sigma).

### Recombinant anosmin-1 protein generation

The human *KAL1* coding sequence was cloned into pCEP-Pu at XbaI/BamHI sites, producing pHis-KAL. The N-terminal 6×His-tagged anosmin-1 protein was generated in 293-EBNA cells, stably transfected with pHis-KAL, cultured in DMEM/F12 (1:1) supplemented with 250 μg/ml G418 and 1 μg/ml puromycin. Cell surface-associated anosmin-1 (Supplementary Figure 1, see section on supplementary data given at the end of this article) was extracted and purified as described by Carafoli *et al*. (2008).

### Motility assay

Nondirectional motility was measured by live time-lapse imaging using an Olympus IX70 inverted microscope (Hamamatsu C4742–95, Hamamatsu, Japan) at 37 °C in 5% CO_2_. The images captured every 15 min were analyzed using ImagePro Plus (Media Cybernetics, Bethesda, MD, USA). The serum-starved cells were incubated with various treatments for 18 h, and cell movements were recorded for further 20 h. The cells were treated with 5% fetal bovine serum (FBS), 50 μM amiloride, 25 μM SU5402, anti-uPA or anti-FGFR1 ectodomain antibodies, and nonspecific mouse IgG (Santa Cruz Biotechnology) at 10 μg/ml. Focal adhesion kinase inhibitor II (PF-228) (Calbiochem, Darmstadt, Germany) was used at 5–20 μM. All inhibitors and vehicle control (DMSO) were added 30 min before anosmin-1 treatment.

### Adhesion assay

Human plasma fibronectin (5 μg/ml) or poly-l-lysine (0.1 mg/ml)-coated culture plates were blocked with 1% BSA. Cells resuspended in Hank's buffered solution containing 2 mM Ca^2^^+^/Mg^2^^+^ were left to adhere for 1 h. The attached cells were stained with 0.25% crystal violet and the absorbance measured at 595 nm.

### Immunofluorescence and BrdU assay

Cells labelled with BrdU for 2 h were fixed and immunofluorescence was performed with anti-BrdU antibody (Millipore, Temecula, CA, USA) using Axiovert 200M fluorescence microscope (Zeiss, Göttingen, Germany). The cells were treated with SU5402 (10 μM), amiloride (25 μM), and recombinant anosmin-1 (20 nM). For the colocalization study, active form of integrin β1 was detected by 12G10 antibody (Millipore) and Alexa Fluor 568 (Molecular Probes, Eugene, OR, USA); EGFP-fused anosmin-1 was detected at 488 nm using an LSM 510 confocal microscope (Zeiss, Jena, Germany). Images were analysed using Zen 2009 Software and ImageJ plugin Wright Cell Imaging Facility.

### Cell proliferation assay

Serum-starved cells were treated with 10 nM recombinant anosmin-1 and the number of viable cells was assessed using CellTitre-Glo Luminescent Cell Viability kit (Promega) following the manufacturer's protocol.

### *KAL1* shRNA

Sequence-verified shRNAs against human *KAL1* (TRCN #73673-73677, Sigma–Aldrich) or nontargeting (nonmammalian) shRNA in pLKO.1-puro lentiviral vector were produced in 293FT packaging cells. A172 cells infected with the viral supernatant were selected in 1 μg/ml puromycin.

### Apoptosis assays

Caspase3/7 activity was assessed using the Caspase-Glo kit (Promega) according to the manufacturer's protocol. To assess the PARP cleavage in western blots, full length PARP was detected by anti-PARP antibody (BD Biosciences, Oxford, UK).

### Western blot and coimmunoprecipitation

Total cell lysate in Triton lysis buffer containing protease/phosphatase inhibitors was analyzed by SDS–PAGE using antibodies against FAK, p-FAK, AKT, p-AKT, ERK, and p-ERK (Cell Signaling, Danvers, MA, USA). Anti-anosmin-1 antibodies were mouse (Novus Biologicals, Littleton, CO, USA) and rabbit polyclonal (LS Biosciences, Seattle, WA, USA). The densitometry values were obtained using Quantity One Software (Bio-Rad). For coimmunoprecipitation, total cell lysates precipitated with anti-β1 integrin (Abcam, Cambridge, UK) or nonspecific IgG (Santa Cruz) were probed using anti-His (Qiagen), anti-GFP (Santa Cruz), or anti-β1 (Cell Signaling) antibodies.

### Zymography

Total cell lysates (0.3% Triton X-100, 50 nM Tris–HCl pH 8.0, 150 nM NaCl with protease inhibitors) and the conditioned medium were collected from serum-starved cells. The samples in loading buffer (10% SDS, 4% glycerol, 0.25 M Tris–HCl pH 6.8, 0.1% bromophenol blue) were loaded on a 10% SDS–polyacrylamide gel copolymerized with 0.1% gelatine A for MMP-2/9 assay or with 2 mg/ml α-casein plus 15 μg/ml plasminogen (Sigma) for uPA assay. The gels incubated in refolding buffer (50 mM Tris–HCl pH 7.4, 100 mM NaCl, 2.5% Triton X-100) and developing buffer (50 mM Tris–HCl pH 7.4, 10 mM CaCl_2_, 0.02% NaN_3_) were stained with 0.1% Coomassie Blue and destained.

### Mouse xenografts

All experiments were in accordance with the local approvals. Female NOD scid gamma (NSG) mice at 6–7 weeks old were purchased from Charles River (Kent, UK) and 3×10^6^ cells in PBS were injected into the flanks. Tumors were measured using callipers and tumor volume (*V*) was estimated by *V*=π×(length (mm))×(width (mm))^2^/6. The tumor doubling time was determined from the slope of a plot of ln(*V*) against time. The paraffin-embedded tumor tissues were analyzed by hematoxylin/eosin staining or anti-Ki67 antibody (Leica Biosystems, UK).

### Statistical analysis

Analysis was carried out using R language and GraphPad Prism 5 Software (La Jolla, CA, USA).

## Results

### *KAL1* is differentially expressed in brain tumor microarrays

To test the notion that anosmin-1 is involved in tumorigenesis of the brain, we examined *KAL1* expression in different types and grades of tumors. We first investigated publicly available microarray data by using meta-analysis. Meta-analysis allows the integration and analysis of heterogeneous datasets. We chose the rank-product method, which has higher sensitivity and selectivity than the conventional *t*-test based methods (Breitling *et al*. 2004, Hong & Breitling 2008). The datasets were selected based on the availability of the histological classification submitted to GEO database (http://www.ncbi.nlm.nih.gov/gds), and were also generated using the Affymetrix U133A (GLP96) gene chip. The samples that did not correspond to the histological typing recognized in the World Health Organization (WHO) grading categories were excluded from the analysis. These criteria limited the sample numbers, at the time of access (October 2009), to a total of 461 samples from ten independent studies, the smallest consisting three samples and the largest 191. The description of tumor types and sample numbers associated with each GEO entry is shown (Table 1). To obtain uniformity of the data, Gene Chip Robust Multi-array Average (GCRMA) normalization was applied to the raw datasets. A total of 19 273 probes were analyzed and the data were then classified into three groups – normal brain, low-grade tumor (WHO grade I and II) and high-grade tumor (WHO grade III and IV). We conducted the meta-analysis on each pair of the three groups – normal (N) vs low-grade (L); low-grade (L) vs high-grade (H); and normal (N) vs high-grade (H) – under the null hypothesis that the paired groups have the same mean expression values. The results show that 33.5, 26.6, and 42.8% of the total probes are differentially expressed (i.e., up- or downregulated) in NL, LH, and NH comparison respectively (Table 2). Notably, in all the three tests, there was a strong evidence (empirical *P* value=0) that *KAL1* is upregulated in both low- and high-grade tumors compared with normal brain, and more elevated in high-grade tumors compared with low-grade ones (Table 3).

Next, among the 9700 probes that showed differential expression, we focused on the 7582 (=3087+2585+573+1337) probes commonly found in at least two meta-analysis tests (Supplementary Figure 2, see section on supplementary data given at the end of this article). We grouped these probes according to four possible expression patterns within each test. The total 12 patterns and the numbers of probes in each category are shown (Supplementary Table 4). Markedly, *KAL1* belonged to the group showing a pattern of N<L<H, indicating a positive correlation of *KAL1* expression with increasing grades.

### Differential expression of *KAL1* in tissue biopsy samples

**Figure 1:**
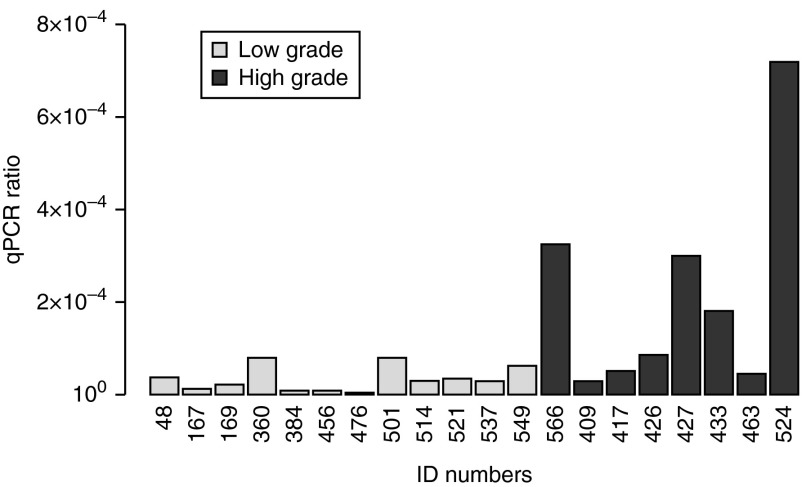
Expression profile of *KAL1* in brain tumor tissues. *KAL1* mRNA levels assessed by qRT-PCR in tumor biopsy samples grouped as low and high grade. Expression ratios represent relative quantification normalized to β-actin. The PCR was performed in triplicates and repeated twice. The average of triplicate samples is shown.

To further verify our findings from the public data analyses, we generated own microarray data from 42 brain tumor biopsy samples obtained at St George's Hospital, which are available in the ArrayExpress database (www.ebi.ac.uk/arrayexpress) under accession number E-MTAB-1852. The cohort included six types of tumors (Table 4). After excluding the ten metastatic adenocarcinomas, the 32 primary tumors were categorized into low (WHO grade I and II, 19 cases) and high (WHO grade III and IV, 13 cases) group based on histopathology. An unpaired *t*-test indicated that *KAL1* was significantly upregulated in high-grade tumors (*P*=4.81×10^−6^, false discovery rate (FDR)=0.256) compared with low-grade tumors. We validated these results by directly assessing *KAL1* mRNA by qRT-PCR. Despite the difficulty of recovering RNA from FFPE tissue samples, the results indicated that high-grade tumors contained higher *KAL1* mRNA compared with low-grade tumors (Welch's *t*-statistic=2.21, *P*=0.031) (Table 4 and Fig. 1). Thus, our gene expression analyses of both public and private datasets support the notion that expression of anosmin-1 correlates with high-grade brain tumors.

### *KAL1* associates with astrocytic tumors

So far, we compared the data purely based on WHO grading regardless of tumor tissue origin. However, in our biopsy samples, 11 out of 13 high-grade tumors were glioblastoma and 15 out of 19 low-grade tumors were meningioma. Therefore, we investigated whether *KAL1* expression correlates with certain tissue origin, regardless of grades. We found a highly significant tendency between different tumor tissue types (one-way ANOVA, *P*=2.20×10^−8^, FDR=8.95×10^−7^) (Table 5). In Tukey–Kramer *post hoc* test, we found *KAL1* was significantly upregulated in tumors of glia/astrocyte origin (e.g., glioblastoma and astrocytoma), compared with tumors of non-glia (e.g., meningioma) or non-brain origin (e.g., adenocarcinoma). Therefore, primary brain tumors of glia/astrocyte origin tend to express high levels of *KAL1* (Table 6).

### *KAL1* expression profiles in the REMBRANDT database

We have also compared the results of *KAL1* analysis available from the REMBRANDT database of the National Cancer Institute (http://rembrandt.nci.nih.gov, data released on 27 July 2010). Compared with normal tissue, *KAL1* was significantly elevated in glioblastoma (*P*=0.0000) for two of the three probe-set analyses available, and was elevated at *P*=0.0392 and *P*=0.0577 in same two probe-sets for astrocytoma and at *P*=0.0019 and 0.0032 for all gliomas. Although REMBRANDT database only uses a categorization for the tissue origin and doesn't include WHO grading system as a component of the analysis, the overall conclusion demonstrated both by us and the REMBRANDT is that *KAL1* is elevated in all tumors compared with nontumor samples and shows a particular association with astrocytoma and glioblastoma. These findings thus support the notion that *KAL1* may contribute to tumor invasion because these tumors exhibit highly infiltrative nature.

### Anosmin-1 increases tumor cell motility

**Figure 2:**
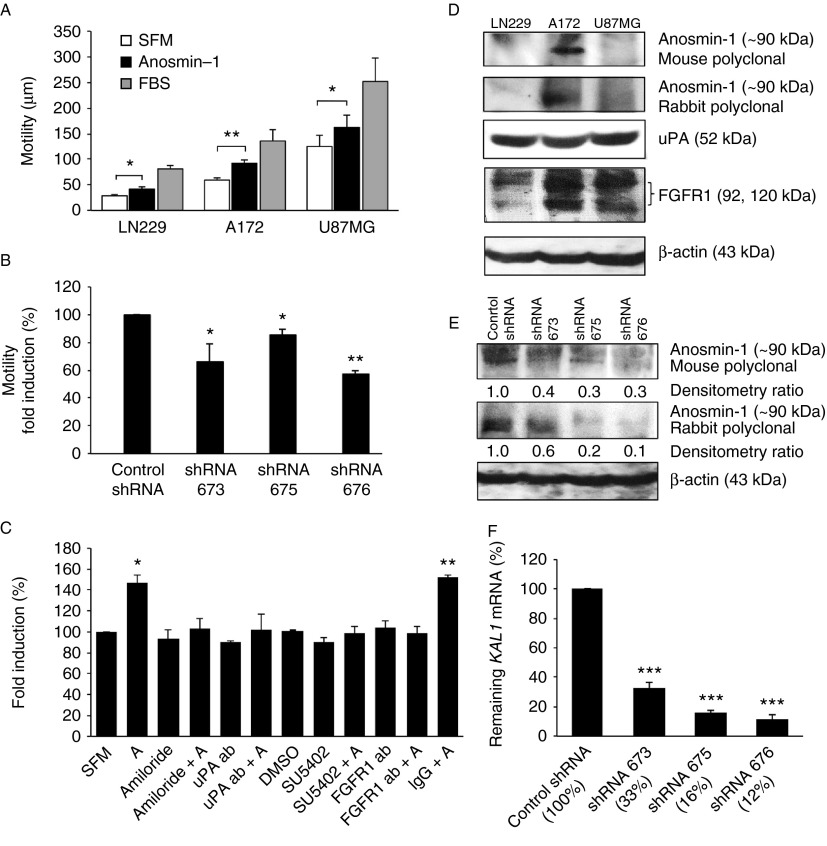
Effects of anosmin-1 in tumor cell motility. (A) Serum-starved cells were treated with either SFM (negative control), 10 nM recombinant anosmin-1, or FBS (positive control). The average moving distance (μm) of 20 random cells tracked over 20 h are shown. Error bars indicate s.e.m. from five independent experiments. The *P* values calculated by two-way ANOVA between the SFM and anosmin-1-treated groups in each cell line are 0.0175 (LN229), 0.0037 (A172), and 0.0399 (U87MG), where *P≤0.05 or **P≤0.01 is considered significant. (B) Effects of *KAL1* knockdown on A172 cell motility. The *P* values obtained from three independent experiments are 0.0395 (for shRNA 673), 0.0253 (for shRNA 675), and 0.0015 (for shRNA 676) when compared with the nontargeting control shRNA. (C) As indicated, LN229 cells were pretreated with chemical inhibitors or specific antibodies for 30 min before addition of anosmin-1 (labeled A). Only anosmin-1 treatment alone or with nonspecific IgG resulted in a significant increase in motility. Error bars indicate s.e.m. from three independent experiments. (D) LN229, A172, and U87MG cells endogenously express anosmin-1, uPA, and FGFR1 proteins at variable levels. See Supplementary Table 3 for the mRNA levels of each gene. (E) *KAL1*-shRNAs significantly knocked down the endogenous anosmin-1 protein as assessed by two different anti-anosmin-1 (mouse or rabbit polyclonal) antibodies. (F) Knockdown efficacy of each shRNA is indicated as the percentage of the remaining *KAL1* mRNA assessed by qRT-PCR, compared with control shRNA, which was significant (***P≤0.0001) in all three shRNAs. qRT-PCR was performed in triplicates, from four independent experiments. Error bars indicate the s.e.m.

As our gene analyses implicated *KAL1* in glioblastomas, we decided to investigate the putative tumor-promoting activities of anosmin-1 *in vitro* using three different glioblastoma cell lines – LN229, A172, and U87MG. The serum-starved cells were treated with recombinant anosmin-1 and the cellular motility was traced using time-lapse microscopy. Anosmin-1 significantly increased cell motility by 50% in LN229, 53% in A172, and 30% in U87MG compared with SFM control, indicating considerable effects of anosmin-1 in multiple glioblastoma cell lines (Fig. 2A). To confirm the specific activity of anosmin-1, we employed lentiviral shRNAs, designated as 673, 675, and 676, in the A172 cells which express a substantial level of endogenous *KAL1* (Fig. 2D and Supplementary Table 3, see section on supplementary data given at the end of this article). After stable infection of A172, the average knockdown of *KAL1* assessed by qRT-PCR was 88% for shRNA676, 84% for shRNA675, and 67% for shRNA673, compared with nontargeting shRNA (Fig. 2F). Western blot analyses using anti-anosmin-1 antibodies confirmed that the reduction in mRNA correlated to the protein suppression in these cells (Fig. 2E). When the shRNA infected cells were examined for motility, all three shRNAs caused a significant decrease (Fig. 2B), indicating the essential role of anosmin-1 in tumor cell motility.

We have previously shown that anosmin-1 directly binds to FGFR1 and uPA, inducing neurite outgrowth, neuronal migration, and proliferation (Gonzalez-Martinez *et al*. 2004, Hu *et al*. 2009). Thus, we asked whether these pathways are operational in tumor cells by examining the anosmin-1-induced motility in the presence of specific inhibitors of FGFR1 (SU5402, FGFR1 ectodomain antibody) or uPA (amiloride, uPA antibody). Pretreatments with these inhibitors abolished the effects of anosmin-1 (Fig. 2C), but not that of FBS (data not shown), suggesting that anosmin-1 induces cell motility through FGFR1- and uPA-dependent mechanisms. Of note, FGFR1 and uPA are expressed in all three cell lines at variable levels (Fig. 2D and Supplementary Table 3).

### Anosmin-1 enhances tumor cell growth

**Figure 3:**
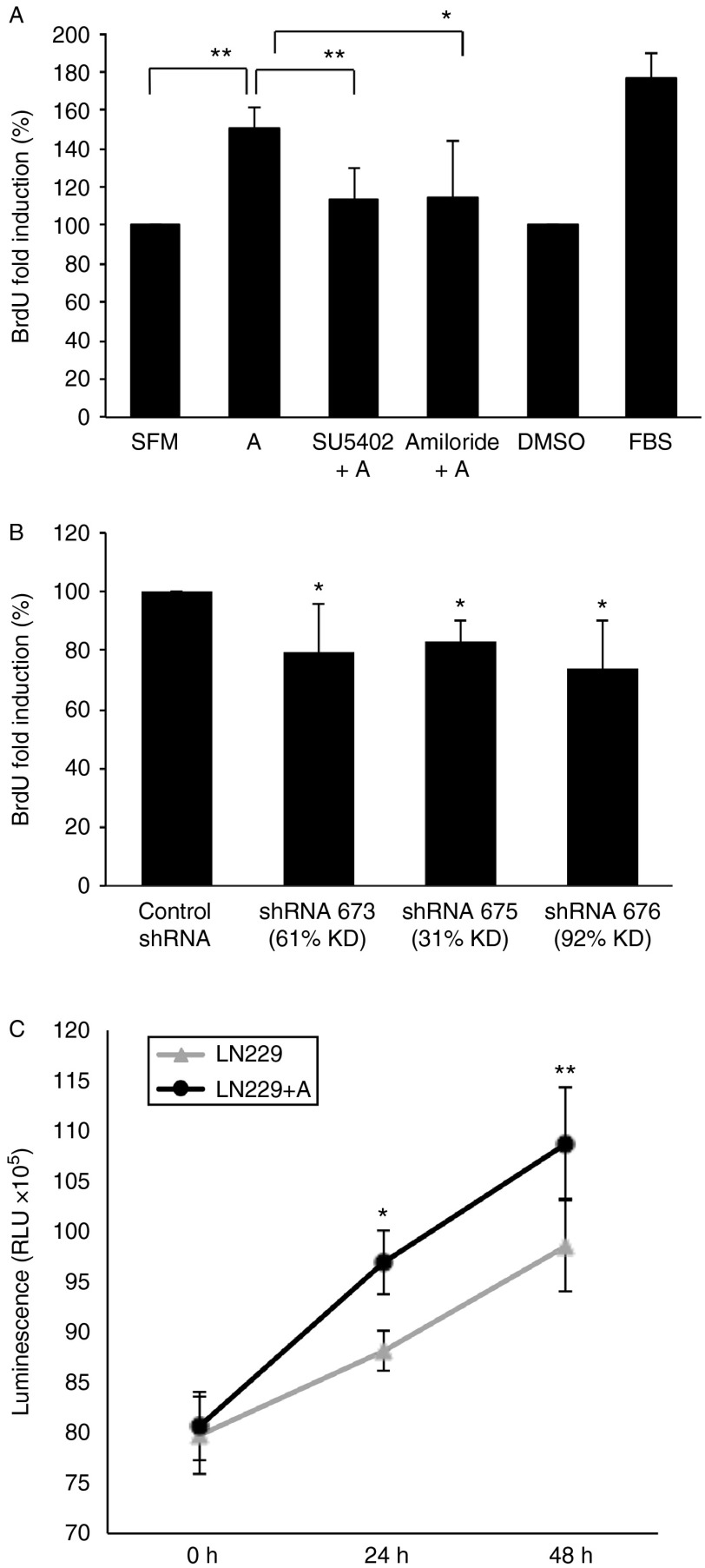
Effects of anosmin-1 in tumor cell proliferation. (A) LN229 cells were treated with anosmin-1 for 6 h with BrdU labeling during the last 2 h in serum-free conditions. In parallel experiments, cells were pre-treated with SU5402 or amiloride for 30 min before stimulation with anosmin-1. Anosmin-1-treated cells (denoted as A) show increased BrdU incorporation (**P=0.0035) compared with the untreated or solvent (DMSO)-treated cells. Pretreatment with SU5402 or amiloride, however, attenuated anosmin-1-induced proliferation (**P=0.0046 and *P=0.0423 respectively). At least five random fields were analysed, which were repeated four times. (B) shRNA-mediated knockdown of *KAL1* results in reduced BrdU incorporation in A172. The *P* values obtained from four independent experiments are 0.0319 (for shRNA673), 0.019 (for shRNA675), and 0.05 (for shRNA676) when compared with the control shRNA. (C) LN229 cells treated with anosmin-1 indicate significantly higher proliferation in CellTitre-Glo luminescent assay, compared with the untreated control at 24 h (*P=0.0103) and 48 h (**P=0.0048) by a two-way ANOVA test. Error bars indicate s.e.m. from five independent experiments.

As high-mitotic index is the hallmark of malignant tumors, we sought to assess the effects of anosmin-1 in glioblastoma cell proliferation by two approaches. We first performed the BrdU incorporation assay in quiescent LN229 cells stimulated with recombinant anosmin-1. There was a 49% increase in the number of cells entering the S phase at 6 h post treatment, which was FGFR1- and uPA-dependent, as it was significantly attenuated by pretreatment with SU5402 or amiloride (Fig. 3A). In contrast, *KAL1* shRNA-infected A172 cells showed a significant reduction in the BrdU incorporation (Fig. 3B). The mitotic effects of anosmin-1 were also assessed based on the number of viable cells in the culture by the ATP quantitation. The LN229 cells cultured in the presence of anosmin-1 showed an increased proliferation compared with the untreated cells at 24–48 h (Fig. 3C).

### Anosmin-1 interacts with β1 integrin complex and induces FAK and AKT signaling

**Figure 4:**
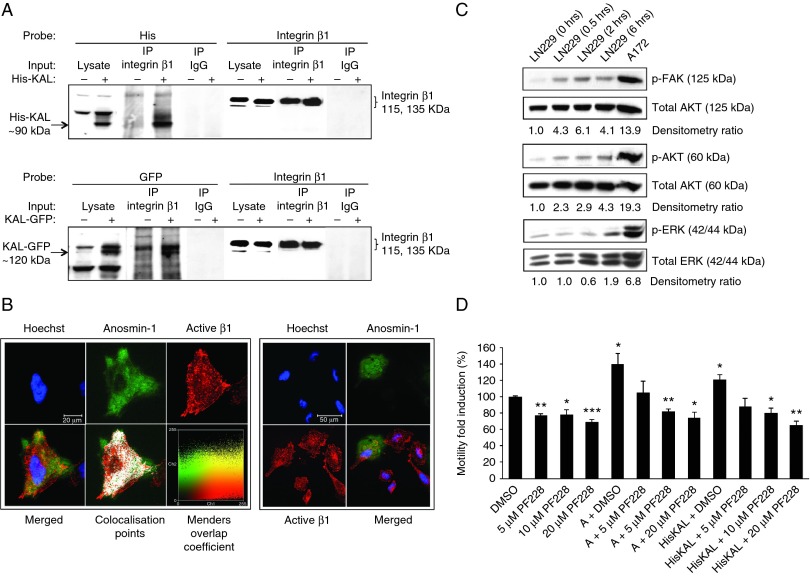
Interaction of anosmin-1 with β1 integrin activates downstream signal pathways. (A) Anosmin-1 co-immunoprecipitated with β1 integrin was identified by probing with anti-His or anti-GFP antibody in LN229 cells transfected with pHis-KAL, pKAL-GFP (+), or empty vector (−). Integrin β1 precipitated by anti-β1 antibody or nonspecific mouse IgG is shown as positive and negative control respectively. (B) Immunofluorescence staining of active integrin β1 (red) in LN229 cells expressing EGFP-tagged anosmin-1 (green). Nuclei were labeled with Hoechst (blue). The colocalization points are also shown (white). A display color-scatter plot is shown of red intensities (Ch1) vs green intensities (Ch2), with the pixels representing the actual color in the image and yellow indicating colocalization. Manders Overlap Coefficient was 0.83, where 1 represents perfect colocalization and 0 represents no colocalization (Manders *et al*. 1992). On the right panel, an independent image demonstrating anosmin-1 localization at the leading edge of a polarized migrating cell. Scale bar is shown. (C) Induction of p-FAK, p-AKT, and p-ERK upon anosmin-1 treatment in serum-starved LN229 cells at the time points indicated. A172 lysate is included as a positive control for constitutive anosmin-1 expression. The ratio of phosphorylated vs total protein determined by densitometry is shown as fold induction compared with the control. All western blots were repeated twice. (D) Effects of FAK inhibitor (PF-228) on anosmin-1-induced motility. Anosmin-1 significantly increased LN229 cell motility (*P=0.0302 in anosmin-1 recombinant protein treated, and *P=0.0208 in HisKAL-transfected). The pretreatment with increasing concentrations of PF-228, but not with the solvent (DMSO), inhibited the effect of anosmin-1 in a dose-dependent manner. PF-228 alone reduced the basal level motility in LN229, as similarly reported in other cancer cell lines (Slack-Davis *et al*. 2007). Error bars indicate s.e.m. from four independent experiments (**, *P*≤0.01; ***, *P*≤0.001).

Both FGFR1 and uPA interact with integrins (Wei *et al*. 1996, Mori *et al*. 2008) and anosmin-1 contains the FNIII domain known to bind to integrins (Ruoslahti 1999). To test whether anosmin-1 regulates integrin function through direct or indirect interaction, we examined the binding of anosmin-1 with β1 integrin that plays major roles in invasive behavior of gliomas and is expressed in all three cell lines we studied (Maglott *et al*. 2006, Brown *et al*. 2008). As shown in Fig. 4A, anosmin-1 was present in the protein complexes precipitated by anti-β1 antibody in *KAL1*-transfected cells, which was identifiable by both N-terminal 6×His- and C-terminal GFP-tagged anosmin-1 constructs, indicating a specific interaction between anosmin-1 and β1, independent of the type or location of tag.

We also conducted a subcellular colocalization study in cells expressing GFP-tagged anosmin-1. Immunofluorescence staining with 12G10 antibody, which labels only the active form of β1, demonstrated that anosmin-1 colocalized with activated β1 with a high Manders Overlap Coefficient. Interestingly, in the polarized migrating cells, anosmin-1 was mainly located at the protruding leading edge but not at the trailing end of the cell (Fig. 4B).

We next asked whether anosmin-1 induces integrin-mediated signal transduction. One of the earliest events upon integrin clustering and signaling is autophosphorylation of FAK at Y397, which leads to the recruitment of PI3K to focal adhesion, causing activation of AKT that regulates integrin-mediated cell survival. Thus, we examined the status of these molecules in LN229 cells at varying time points after anosmin-1 treatment. The results show that anosmin-1 induced phosphorylation of FAK within 30 min, which peaked at around 2 h. The phosphorylated AKT also appeared within 30 min, gradually increasing up to 6 h. Similarly, phosphorylation of ERK gradually increased for up to 6 h. Notably, A172 cells which express a substantial level of endogenous anosmin-1 exhibited high levels of phosphorylation of these molecules even in SFM (Fig. 4C). To confirm the involvement of these pathways in anosmin-1 function, we examined the effects of a FAK inhibitor PF-228 on the anosmin-1-induced motility. It has been reported that PF-228 inhibits FAK phosphorylation (Tyr^397^) and FAK-mediated random motility in multiple cancer cell lines (Slack-Davis *et al*. 2007). In LN229 cells, PF-228 reduced the basal level of motility about 20% in SFM. Importantly, the increasing concentration of PF-228 abolished the anosmin-1-induced motility in a dose-dependent manner (Fig. 4D), supporting that FAK signaling is required for anosmin-1 function. Combined, these data demonstrate that anosmin-1 interacts with integrins, inducing downstream FAK, AKT, and ERK signaling events.

### Effects of anosmin-1 in integrin-mediated cell adhesion and apoptosis

**Figure 5:**
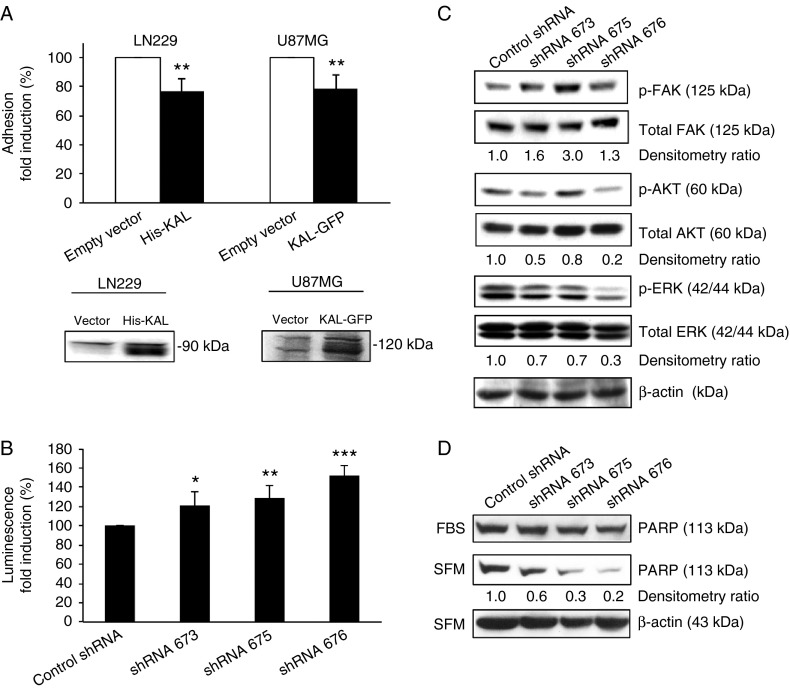
Effects of anosmin-1 on cell adhesion and survival. (A) The percentage of cells adhered to the fibronectin-coated plates after 1 h at 37 °C was quantified and normalized to the nonspecific total adhesion on the poly-l-lysine-coated plate. LN229 His-KAL cells show 23% reduction (**P=0.0044) and U87MG KAL-GFP show 21% reduction (**P=0.0011) in cell adhesion, compared with the empty vector control. Experiments were performed in quintuplicate and repeated five times. Expression of the transfected anosmin-1 constructs is confirmed by anti-His or anti-GFP antibodies. (B) Effects of *KAL1* knockdown on apoptosis. Caspase3/7 activity of A172 cells infected with shRNA was measured in relative light units and the average fold induction from four independent experiments is shown. *P* values are 0.0504 (for shRNA 673), 0.0086 (for shRNA 675), and 0.0002 (for shRNA 676). (C) Effects of *KAL1* knockdown in phosphorylation status of FAK, AKT, and ERK in A172 cells. The relative ratio of phosphorylated vs total protein assessed by densitometry is shown as fold induction compared with the control shRNA, normalized to β-actin loading control. (D) Induction of PARP protein cleavage by *KAL1* knockdown. The full length PARP protein is assessed by western blot in shRNA-infected A172 cells before (FBS) and after serum-starvation (SFM). The densitometry ratio is shown as fold induction compared with the control shRNA, normalized to the β-actin loading controls.

The Arg-Gly-Asp (RGD) sequence motif present in FNIII repeats of several ECM proteins including fibronectin is known as the integrin-binding site, and peptides that contain such motif compete with fibronectin for integrin binding, thereby inhibiting the attachment of the cells to a fibronectin matrix (Ruoslahti 1996). We hypothesize that anosmin-1 which also contains multiple FNIII domains may compete with fibronectin, modulating integrin-mediated cell adhesion. To test this notion, we performed a cell adhesion assay on fibronectin-coated plates using LN229 and U87MG cells. Anosmin-1 expressing cells adhere significantly less to the fibronectin surface compared with the control (Fig. 5A), suggesting that anosmin-1 potentially alters integrin-mediated cell adhesion on fibronectin-containing matrix.

As anosmin-1 modulates cell adhesion (Fig. 5A) and activates prosurvival signals (Fig. 4C), we speculate that loss of anosmin-1 may induce apoptosis. First, we assessed caspase3/7 activity in shRNA-infected cells after 24 h serum-starvation. All three *KAL1*-knockdown lines showed significant increases in caspase3/7 activity, in accordance with the knockdown efficiency indicating specific effects of anosmin-1 (Fig. 5B). Then the cleavage of poly (ADP-ribose) polymerase (PARP) was investigated to further assess apoptosis. Compared with the controls, *KAL1*-knockdown cells contained significantly reduced levels of the full-length PARP protein, suggesting enhanced cleavage (Fig. 5D). We then asked whether this induction of apoptosis is accompanied by reduced phosphorylation of AKT and ERK (Fig. 5C). We observed 80% decrease in AKT phosphorylation and 70% decrease in ERK phosphorylation by shRNA676 with 88% knockdown of *KAL1*. However, *KAL1*-knockdown did not show consistent reduction in FAK phosphorylation (Fig. 2B), indicating a functional redundancy of anosmin-1 in FAK activation at the focal adhesion site. As A172 contains relatively high levels of endogenous FAK/p-FAK compared with other GBM cell lines such as LN229 (Liu *et al*. 2007), multiple pathways leading to FAK phosphorylation might be operational in A172.

### Anosmin-1 induces extracellular protease activities

**Figure 6:**
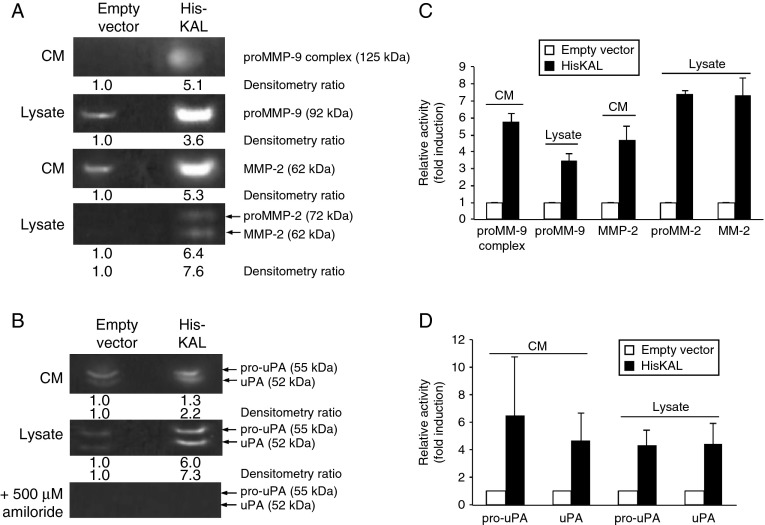
Anosmin-1 induces MMP2/9 and uPA proteolytic activity. Empty vector and pHis-KAL-transfected LN229 were subjected to gelatin zymography to assess MMP2 and MMP9 activities (A) or plasminogen zymography to assess uPA activity (B). Molecular sizes of respective proteins are shown in brackets. The 125 kDa band was previously reported as the heterodimer of proMMP9 with lipocalin (Yan *et al*. 2001, Rorive *et al*. 2008). The bands in the plasminogen zymography were inhibited by amiloride treatment, confirming the specificity of the assay. Each lane was loaded with either 40 μl conditioned medium (CM) or 80 μg total cell lysates. The fold inductions of enzyme activity are indicated in relation to the vector transfected cells, as determined by densitometry of the respective bands in inverted images. (C and D) The average fold induction of the relative band intensity is shown as a graph. All experiments were repeated three times. Error bars indicate s.e.m.

As increased levels of extracellular proteases such as uPA and matrix metalloproteinases (MMPs) have a strong correlation with glioma cell motility and invasion (Gladson *et al*. 1995, Forsyth *et al*. 1998), we postulated that anosmin-1 might modulate these proteases. The presence and activities of MMP2 and MMP9 were previously described in LN229 cells (Yan *et al*. 2001, Rorive *et al*. 2008, Nan *et al*. 2010). Thus, we conducted the gelatin zymography on LN229 with or without *KAL1* transfection. We detected both the pro- and active forms of MMP2 but only the proform of MMP9 in the cell lysates and conditioned medium. However, their activities were significantly increased (three- to seven-fold) in *KAL1*-transfected cells (Fig. 6A). As uPA regulates MMP activities, we also asked whether anosmin-1 affects uPA in the plasminogen zymography. The *KAL1*-transfected cells showed three- to six-fold increase in both pro- and active forms of uPA (Fig. 6B). Therefore, anosmin-1 increases the proteolytic activities of MMP2/9 and uPA in glioblastoma cells, which may contribute to the ECM remodeling during tumor invasion.

### Anosmin-1 enhances tumor growth in mouse xenograft model

**Figure 7:**
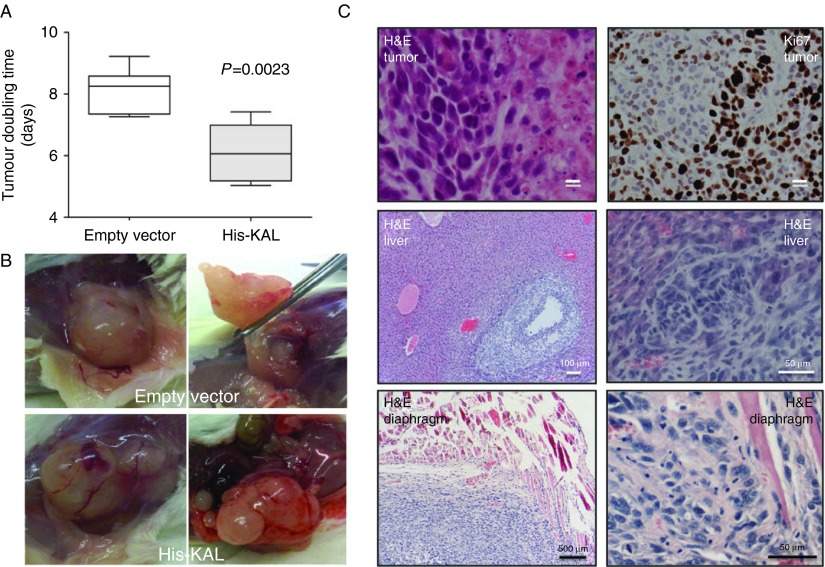
Anosmin-1 promotes tumor growth *in vivo*. (A) Tumor volume (*V*) was calculated using the formula, *V*=π×(length (mm))×(width (mm))^2^/6. Tumor doubling time was generated from the slope of a plot of ln(*V*) against time (days post transplantation). Mean tumor doubling times were 8.12±0.72 days for the control group and 6.11±0.98 days for the LN229 His-KAL group (*P*=0.0023, two-tailed *t*-test). (B) Images of a xenograft tumor grown in the NSG mice. This His-KAL tumor showed prominent vasculature and attachment to the surrounding tissues. (C) Hematoxylin and eosin (H&E) staining of the tumour tissue indicates the characteristics of malignant glioblastoma, such as pleomorphic cells, mitoses, and areas of necrosis, with a high Ki67 score (Supplementary Table 4) in both groups. Infiltration of the His-KAL tumor into the liver and diaphragm is identified by H&E staining.

Our *in vitro* data encouraged us to further investigate the role of anosmin-1 *in vivo*. Therefore, we transplanted LN229 cells with or without *KAL1*-overexpression into the flanks of immunodeficient mice. Palpable tumors were detected after 2–3 weeks. At 53–70 days post-implantation when the tumors reached ∼500 mm^3^ in volume, they were excised and embedded in paraffin for histological analysis. Tumors from both groups exhibited the histological characteristics of malignant tumors such as pleomorphic cells, necrosis, and active mitoses with high Ki67 scores (Fig. 7C and Supplementary Table 4), but those derived from the *KAL1*-transfected cells grew significantly faster compared with the empty vector-transfected tumors (Fig. 7A). Mean tumor doubling times were 8.12±0.72 days for the control group and 6.11±0.98 days for the His-KAL group (*P*=0.0023). Upon autopsy of the mice, we observed that two out of the six anosmin-1-expressing tumors showed much prominent gross vasculature and physical attachment to the adjacent organs (Fig. 7B). Further histological analysis of these organs indicated the presence of infiltrating tumors within the liver and diaphragm (Fig. 7C). The continued expression of the transfected *KAL1* in the His-KAL tumor tissues was confirmed by qRT-PCR (Supplementary Figure 3, see section on supplementary data given at the end of this article). These results demonstrate that overexpression of anosmin-1 may associate with the aggressive growth of tumors *in vivo*.

## Discussion

Reappearance of an embryonic pattern of ECM has been observed in pathological conditions in adulthood (Ffrench-Constant *et al*. 1989, Coutu *et al*. 2011) and aberrant expression of developmental genes results in malignant brain tumors (Goodrich *et al*. 1997). Here anosmin-1, whose function was implicated mainly in the early migratory events of GNRH neurons in the developing brain, is shown to be upregulated in the primary brain tumors, while it remains at low levels in normal brain (Tables 1, 2 and 3). The majority of glioblastoma multiforme (GBM), the most fatal and common brain tumor, is primary (*de novo* development), but some GBMs develop from lower grade astrocytomas. *KAL1* expression associated with not only the grade of tumor but also the tissue type, showing relations with glia and astrocyte origin, but not in the metastatic tumors of other tissue types (Tables 5 and 6). In fact, *KAL1* is reported to be downregulated in colon, lung, and ovarian cancer compared with normal tissues (Jian *et al*. 2009). It is unknown whether *KAL1* is expressed in glia or astrocytes during embryogenesis. However, glia cells play fundamental roles in neuronal pathfinding, thus the failed GNRH neuronal migration in KS could be caused by the primary glial defects.

The infiltration of neoplastic cells into the surrounding healthy brain is the main source for recurrence and failure of current treatment. Anosmin-1 enhanced glioblastoma cell motility (Fig. 2) and colocalized with the leading edge of migrating cells (Fig. 4B) while enhancing uPA and MMP2/9 activities (Fig. 6). It is conceivable that anosmin-1 concentrates these proteases at the leading edge of the developing tumor, contributing to their infiltration. Although the xenograft models showed evidence of invasion in the presence of anosmin-1 (Fig. 7), the microenvironment of the brain parenchyma is different from that of flanks, thus the effects of anosmin-1 in brain tumor invasion will need to be reconfirmed in orthotopic models.

The 32 amino acid sequences within the first FNIII domain of anosmin-1 was previously reported to serve as an adhesion matrix for different neuronal and non-neuronal cell types *in vitro* (Soussi-Yanicostas *et al*. 1998, Bribian *et al*. 2008), but the molecular basis for this was not understood. We now postulate that the differential adhesion to anosmin-1 may depend on the repertoire of integrins expressed on the surface of these cells. Anosmin-1 does not contain the typical integrin recognition motif, RGD. Nonetheless, numerous variations which do not resemble RGD but are capable of binding to different integrins have been described (Ruoslahti 1996). Although the identity of the integrin α subtypes involved in anosmin-1 interaction is not clear, human GBM tissues exhibit strong expression of α2β1, α5β1, and α6β1, compared with normal brain (Gingras *et al*. 1995). Furthermore, α9β1 was shown to regulate LN229 cell proliferation and survival (Brown *et al*. 2008) and α5β1 antagonist inhibited adhesion and proliferation in A172 and U87MG cells (Maglott *et al*. 2006).

Both uPA and MMPs are upregulated in malignant brain tumors (Gladson *et al*. 1995). FGFR1 is reported to be mutated in glioblastomas (Rand *et al*. 2005). Currently, it is unclear whether anosmin-1 directly binds to integrin or involves other interactants. We showed that anosmin-1 may protect cells from apoptosis (anoikis), while facilitating detachment and migration. The source of anti-apoptotic signals for the migrating GNRH neurons during development has not been identified. Although Gas6 and its receptor Axl signaling has been suggested (Allen *et al*. 1999), Gas6/Axl is not expressed in the GNRH neurons themselves.

In summary, the loss-of-function mutations of anosmin-1 disrupt the normal GNRH neuronal development, thus causing KS, but inappropriate activation of anosmin-1-mediated signal pathways in the developed brain is the underlying mechanism of some malignant brain tumors.

## Supplementary data

This is linked to the online version of the paper at http://dx.doi.org/10.1530/ERC-13-0181.

Supplementary Data

## Figures and Tables

**Table 1:** Meta-analysis of public microarray data. Microarray samples (total 461) from GEO database used in the meta-analyses

**Group**	**GEO series ID**	**Number and types of selected samples**
Normal brain (53)	GSE12649	34 (adult postmortem brain)
	GSE5389	11 (adult postmortem brain)
GSE5390	8 (adult postmortem brain)
Low grade tumor (32)	GSE12907	21 (grade I astrocytoma, pilocytic)
	GSE3185	3 (grade I and II astrocytoma)
	GSE4780	5 (grade I and II meningioma)
GSE8692	3 (oligodendroglioma, mixed glioma)
High grade tumor (376)	GSE4271	100 (grade III and IV astrocytoma)
GSE13041	191 (glioblastoma multiforme)
	GSE4412	85 (glioblastoma multiforme, anaplastic astrocytoma, anaplastic mixed oligo-astrocytoma, anaplastic oligodendro-glioma)

**Table 2:** Meta-analysis of public microarray data. Differentially expressed (DE) probes at 0.001 significance level in the three meta-analyses

**Tests**	**No. of significant DE probes** [alternative hypothesis]		**Total no. of DE probes** (genes)	**Percentage of DE probes** (DE probes/total probes)
Normal vs low (NL)	3213 [N<L]	3257 [N>L]	6470 (4864)	33.5% (=6470/19 273)
Low vs high (LH)	3008 [L<H]	2124 [L>H]	5132 (4034)	26.6% (=5132/19 273)
Normal vs high (NH)	4706 [N<H]	3560 [N>H]	8266 (6072)	42.8% (=8266/19 273)

The alternative hypothesis is shown in brackets.

**Table 3:** Meta-analysis of public microarray data. Statistics of *KAL1* in three meta-analyses

**Tests**	**Rank-product statistic** (empirical *P* value) [alternative hypothesis]	
Normal vs low (NL)	2637.756 (*P*<0.01) [N<L]	9955.508 (*P*=0.993) [N>L]
Low vs high (LH)	3992.405 (*P*<0.01) [L<H]	5779.429 (*P*=0.072) [L>H]
Normal vs high (NH)	2375.587 (*P*<0.01) [N<H]	12 815.553 (*P*=1.000) [N>H]

The lower the rank-product value is, the more significantly different the *KAL1* expression is in each test. Alternative hypothesis is shown in brackets.

**Table 4:** Analysis of 42 brain tumor cases from St George's Hospital

**Sample ID**	**Sex**	**Age**	**KAL1 level by microarray**	**KAL1 level by qRT-PCR**	**Type of tumor**
524	M	34	9.57	7.17×10^−4^	Glioblastoma (WHO grade IV)
427	M	59	11.79	2.98×10^−4^	Glioblastoma (WHO grade IV)
433	F	70	11.88	1.77×10^−4^	Glioblastoma (WHO grade IV)
409	M	57	11.13	2.67×10^−5^	Glioblastoma (WHO grade IV)
426	F	61	10.97	8.31×10^−5^	Glioblastoma (WHO grade IV)
417	M	56	11.48	4.87×10^−5^	Glioblastoma (WHO grade IV)
463	F	65	9.88	4.15×10^−5^	Glioblastoma (WHO grade IV)
018	M	79	10.14	NA	Glioblastoma (WHO grade IV)
461	M	16	11.26	NA	Glioblastoma (WHO grade IV)
369	F	65	10.42	NA	Glioblastoma (WHO grade IV)
460	F	58	9.62	NA	Glioblastoma (WHO grade IV)
366	M	40	9.96	3.23×10^−4^	Anaplastic ependyoma (WHO grade III)
140	F	27	11.08	NA	Anaplastic astrocytoma (WHO grade III)
079	F	40	12.50	NA	Astrocytoma (WHO grade II)
403	M	19	10.28	NA	Gemistocytic astrocytoma (WHO grade II)
360	M	49	3.94	7.73×10^−5^	Meningioma (WHO grade I)
501	F	71	8.95	7.72×10^−5^	Meningioma (WHO grade I)
549	F	66	6.55	5.96×10^−5^	Meningioma (WHO grade I)
048	F	78	5.73	3.39×10^−5^	Meningioma (WHO grade I)
521	F	88	7.77	3.28×10^−5^	Meningioma (WHO grade I)
514	F	46	7.56	2.74×10^−5^	Meningioma (WHO grade I)
169	M	66	8.00	1.90×10^−5^	Meningioma (WHO grade I)
167	F	35	6.99	9.98×10^−6^	Meningioma (WHO grade I)
456	M	52	7.08	4.14×10^−6^	Meningioma (WHO grade I)
384	M	49	5.35	3.89×10^−6^	Meningioma (WHO grade I)
476	F	65	7.57	0 (non-detected)	Meningioma (WHO grade I)
455	F	60	8.89	NA	Meningioma (WHO grade I)
367	F	37	3.92	NA	Meningioma (WHO grade I)
394	F	62	7.23	NA	Meningioma (WHO grade I)
502	M	72	3.18	NA	Meningioma (WHO grade I)
075	M	20	11.34	NA	Pilocytic astrocytoma (WHO grade I)
537	F	71	6.76	2.64×10^−5^	Haemangioblastoma (WHO grade I)
449	M	70	6.10	2.68×10^−4^	Metastatic adenocarcinoma
408	M	67	4.66	4.64×10^−5^	Metastatic adenocarcinoma
210	F	69	7.91	1.44×10^−5^	Metastatic adenocarcinoma
153	F	58	9.91	4.20×10^−5^	Metastatic adenocarcinoma
536	M	73	5.33	1.43×10^−5^	Metastatic adenocarcinoma
166	F	56	6.14	NA	Metastatic adenocarcinoma
475	M	60	8.13	NA	Metastatic adenocarcinoma
398	F	73	8.78	NA	Metastatic adenocarcinoma
211	F	65	9.22	NA	Metastatic adenocarcinoma
438	M	61	6.99	NA	Metastatic large cell carcinoma

The patients, types of tumors, and *KAL1* expression levels assessed by microarray and qRT-PCR are shown. *KAL1* mRNA level determined by qRT-PCR is indicated as the concentration ratio normalized to β-actin (shown in Fig. 1); NA, not determined due to limited tissue availability.

**Table 5:** Differential expression of *KAL1* in different tissue origin. Microarray mean expression value of *KAL1* gene in each tumor type is shown in this table

**Tissue**	**Meningioma**	**Astrocytoma**	**Glioblastoma**	**Adenocarcinoma**
Mean (s.d.)	7.32 (1.75)	11.30 (0.85)	10.74 (0.85)	6.58 (1.79)

**Table 6:** Differential expression of *KAL1* in different tissue origin analyzed by Tukey's test

	**Adenocarcinoma**	**Astrocytoma**	**Glioblastoma**	**Meningioma**
Adenocarcinoma	–	**3.98 (4.4**×**10**^**−4**^**)**	**3.42 (5.0**×**10**^**−5**^**)**	−0.74 (0.633)
Astrocytoma	–	–	0.56 (0.92)	**−4.72 (1.6**×**10**^**−5**^**)**
Glioblastoma	–	–	–	**−4.16** **(2.0**×**10**^**−6**^**)**
Meningioma	–	–	–	–

Each value represents the mean expression difference between two tumor types (column vs row). *P* value is shown in the bracket and only the significant results (*P* value <0.01) are in bold.
