# Supplementary material for: Anosmin-1 contributes to brain tumor malignancy through integrin signal pathways
Source: Endocr Relat Cancer. 2013 Nov 4;21(1):85–99. doi: 10.1530/ERC-13-0181 (PMC3869950; doi:10.1530/ERC-13-0181)
Supplement: Supplementary Data [file supp_21_1_85__index.html]

Supplementary Data 

# Anosmin-1 contributes to brain tumor malignancy through integrin signal pathways

## Supplementary Data

**Files in this Data Supplement:**

- Supplementary Table 1 - Primer sequences and annealing temperatures used in the PCR reactions. (PDF 53 KB)
- Supplementary Table 2 - **Twelve expression patterns of differential probes**. The expression patterns of 7582 probes (identified from supplementary Fig.2) in the meta-analysis. Total 12 patterns and the probe numbers are shown. The + or - sign represents up- or down-regulation, respectively, in comparison to their base group denoted as B. (e.g. 2806 probes in pattern 1 are significantly over-expressed in the low group by the *NL* test and, at the same time, they are up-regulated in the high group by the *NH* test.) *KAL1* belongs to pattern 1, 5 and 9 (shown in bold). (PDF 115 KB)
- Supplementary Table 3 - Relative mRNA levels of different genes in the cell lines used in the study. (PDF 99 KB)
- Supplementary Table 4 - **Ki67 values of the xenograft tumor tissues** Paraffin-embedded tissue sections of xenograft tumors (6 per group) were analysed by immunohistochemistry using anti-Ki67 antibody. Average Ki67 score was calculated from 15 random fields. (PDF 48 KB)
- Supplementary Figure 1 - **Generation of purified recombinant anosmin-1 protein**. (A) Anosmin-1 protein from the salt extract of pHisKAL-transfected 293EBNA cells was purified as similarly described (Carafoli *et al*. 2008). The purity of the eluted protein and the concentrated preparation is shown on SDS-PAGE gel stained with Coomassie Brilliant Blue. (B) Western blot analysis using anti-His antibody indicates the presence of recombinant anosmin-1 protein in the conditioned medium and salt extract of the pHisKAL-transfected (+), but not in the empty vector transfected (-) cells. (PDF 167 KB)
- Supplementary Figure 2 - **The number of differentially expressed probes identified from the *NL*, *NH*, and *LH* tests**. Total 9700 differential probes were identified and 7582 (=3087+2585+573+1337) probes which were commonly detected in at least two tests were chosen for further analyses as shown in Table 1C. (PDF 371 KB)
- Supplementary Figure 3 - **Expression of *KAL1* in the xenograft tissues**. The xenograft tumors derived from the LN229 cells transfected with either His-KAL or empty vector were snap frozen, cryo-sectioned and analyzed by qRT-PCR. Continued expression of the transfected *KAL1* in different regions of the tumor was confirmed from RNA samples extracted from the core, middle and edge of the tumors. Tumors derived from the empty vector transfected LN229 did not show any *KAL1* expression. Mean expression ratios of each group (N=6) are shown. Data were normalized to β-actin and qRT-PCR was performed in triplicates. Error bars indicate the S.E.M. (PDF 48 KB)
