# Supplementary material for: Anosmin-1 contributes to brain tumor malignancy through integrin signal pathways
Source: Endocr Relat Cancer. 2013 Nov 4;21(1):85–99. doi: 10.1530/ERC-13-0181 (PMC3869950; doi:10.1530/ERC-13-0181)
Supplement: Supplementary Data [file supp_ERC-13-0181_Supplementary_table_1.pdf]

Table S1. Primer sequences and annealing temperatures used in the PCR reactions

β-Actin forward	5'AGA GCT ACG AGC TGC CTG AC3'	60°C
β-Actin reverse	5'AGC ACT GTG TTG GCG TAC AG3'	
KAL1 forward	5'CCT TCT TCC AGC ACT TCC AG 3'	60 ^{o}C
KAL1 reverse	5'TCA CAG CTG GTC AAG CAT TC 3'	
uPA forward	5'TGA GGT GGA AAA CCT CAT CC 3'	58 ^{o}C
uPA reverse	5' GGC AGG CAG ATG GTC TGT AT 3'	
FGFR1 forward	5'CTG GAT GTC GTG GAG CGG TCC 3'	65 ^{o}C
FGFR1 reverse	5' TTC CAG AAC GGT CAA CCA TGC AGA 3'	
pHisKAL, forward	5'GCG CTA GCC GCG GCC GGC CCC GGC GCG GCT3'	50 ^{o}C
pHisKAL, reverse	5'CCG GAT CCT TAG TAT CTT TCT GGA GAA GG3'	
