# Supplementary material for: Anosmin-1 contributes to brain tumor malignancy through integrin signal pathways
Source: Endocr Relat Cancer. 2013 Nov 4;21(1):85–99. doi: 10.1530/ERC-13-0181 (PMC3869950; doi:10.1530/ERC-13-0181)
Supplement: Supplementary Data [file supp_ERC-13-0181_Supplementary_table_2.pdf]

Table S2. Twelve expression patterns of differential probes. The expression patterns of 7582 probes (identified from supplementary Fig.2) in the meta-analysis. Total 12 patterns and the probe numbers are shown. The + or – sign represents up- or down-regulation, respectively, in comparison to their base group denoted as B (e.g. 2806 probes in pattern 1 are significantly over-expressed in the low group by the *NL* test and, at the same time, they are up-regulated in the high group by the *NH* test). *KAL1* belongs to pattern 1, 5 and 9 (shown in bold).

Pattern	Normal	Low	High	No. probes
**1**	**B**	**+**	**+**	**2806**
2	B	+	-	54
3	B	-	+	45
4	B	-	-	2767
**5**	**-**	**B**	**+**	**592**
6	-	B	-	1098
7	+	B	+	1063
8	+	B	-	405
**9**	**-**	**-**	**B**	**1629**
10	-	+	B	759
11	+	-	B	740
12	+	+	B	794
