# Supplementary material for: Anosmin-1 contributes to brain tumor malignancy through integrin signal pathways
Source: Endocr Relat Cancer. 2013 Nov 4;21(1):85–99. doi: 10.1530/ERC-13-0181 (PMC3869950; doi:10.1530/ERC-13-0181)
Supplement: Supplementary Data [file supp_ERC-13-0181_Supplementary_table_3.pdf]

Table S3. Relative mRNA levels of different genes in the cell lines used in the study.

The crossing point (CP) of each sample in triplicates was normalized to the β-actin control and the expression ratio was calculated using the equation, $\mathrm{Ratio} = \frac{E^{CP_{\beta-actin}}}{E^{CP_{KAL1}}}$ where E stands for efficiency of the reaction which was approximately 2.

	**KAL1**	**uPA**	**FGFR1**
**LN229**	0	5.51E-05	8.18E-03
**A172**	2.54E-05	3.52E-04	2.00E-02
**U87MG**	0	5.00E-03	1.00E-02
