# Supplementary material for: Anosmin-1 contributes to brain tumor malignancy through integrin signal pathways
Source: Endocr Relat Cancer. 2013 Nov 4;21(1):85–99. doi: 10.1530/ERC-13-0181 (PMC3869950; doi:10.1530/ERC-13-0181)
Supplement: Supplementary Data [file supp_ERC-13-0181_Supplementary_table_4.pdf]

Table S4. Ki67 values of the xenograft tumor tissues.

Paraffin-embedded tissue sections of xenograft tumors (6 per group) were analysed by immunohistochemistry using anti-Ki67 antibody. Average Ki67 score was calculated from 15 random fields.

Cell line	Mouse ID	Ki67 score	Std Dev
**Empty vector**	1	71.61	6.57
	2	71.59	12.80
	3	50.67	9.54
	4	49.00	10.04
	5	51.39	11.37
	6	56.19	11.55
**His-KAL**	1	63.67	9.72
	2	78.01	8.16
	3	52.17	10.52
	4	50.71	13.94
	5	49.69	14.61
	6	45.72	13.01
