# Supplementary figures and images for: Anosmin-1 contributes to brain tumor malignancy through integrin signal pathways
Source: Endocr Relat Cancer. 2013 Nov 4;21(1):85–99. doi: 10.1530/ERC-13-0181 (PMC3869950; doi:10.1530/ERC-13-0181)

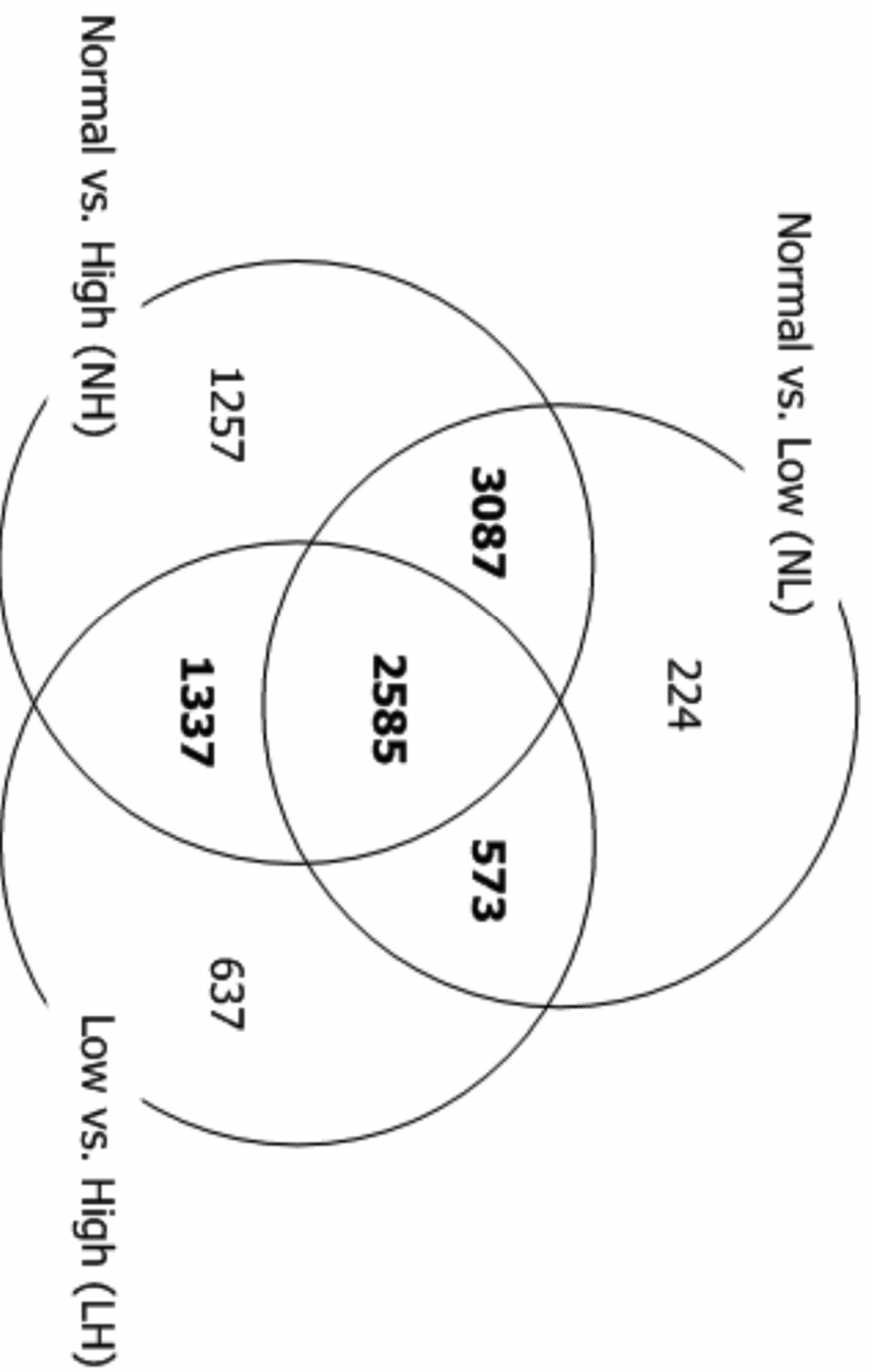

Supplement: Supplementary Data [file supp_ERC-13-0181_Supplementary_figure_1.pdf]

**A**

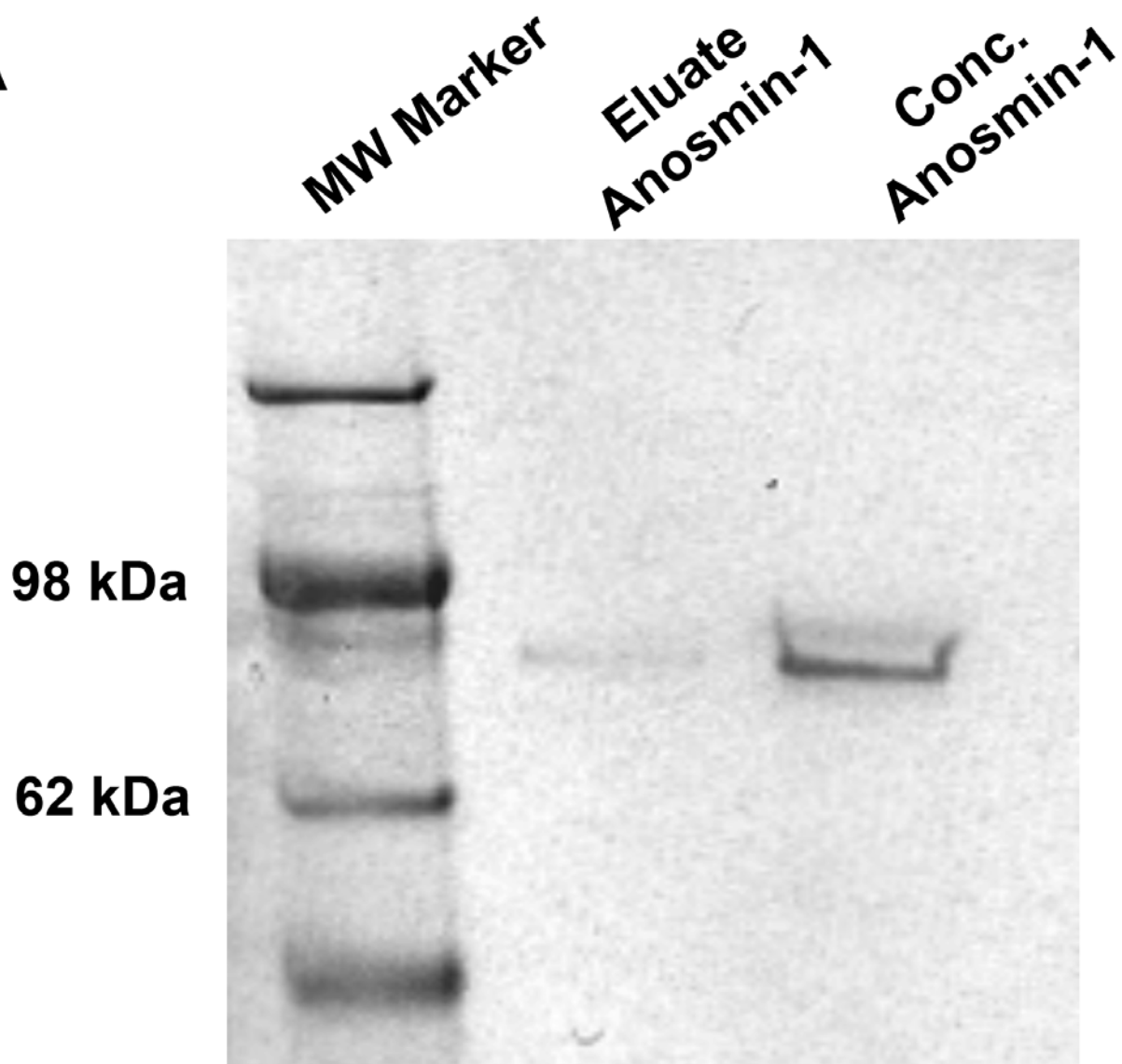

**B**

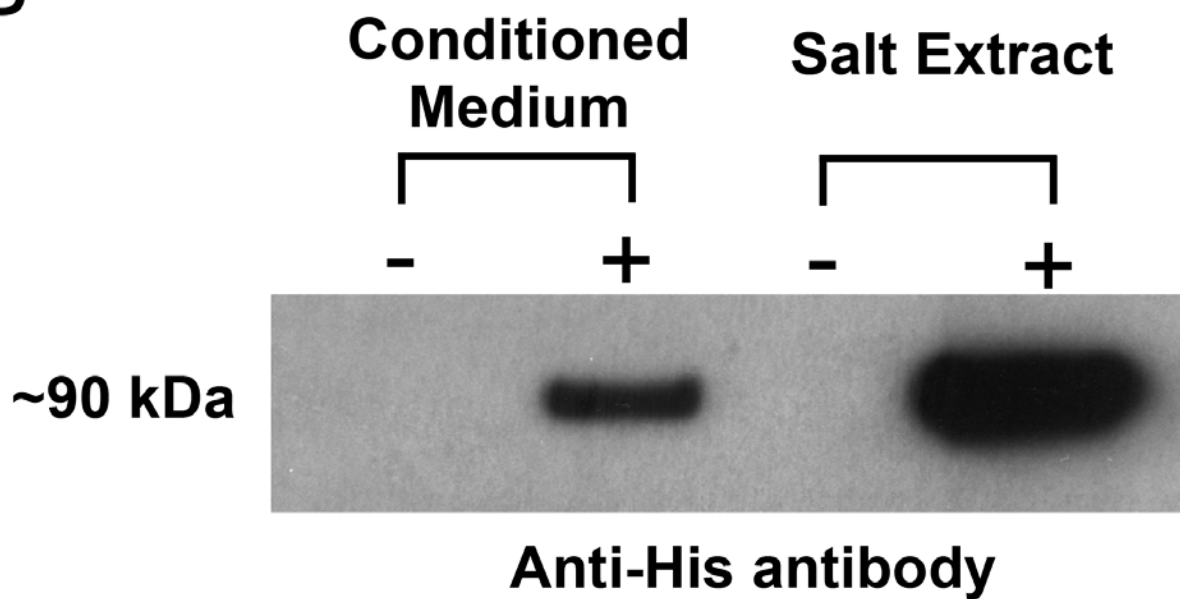

Supplement: Supplementary Data [file supp_ERC-13-0181_Supplementary_figure_2.pdf]

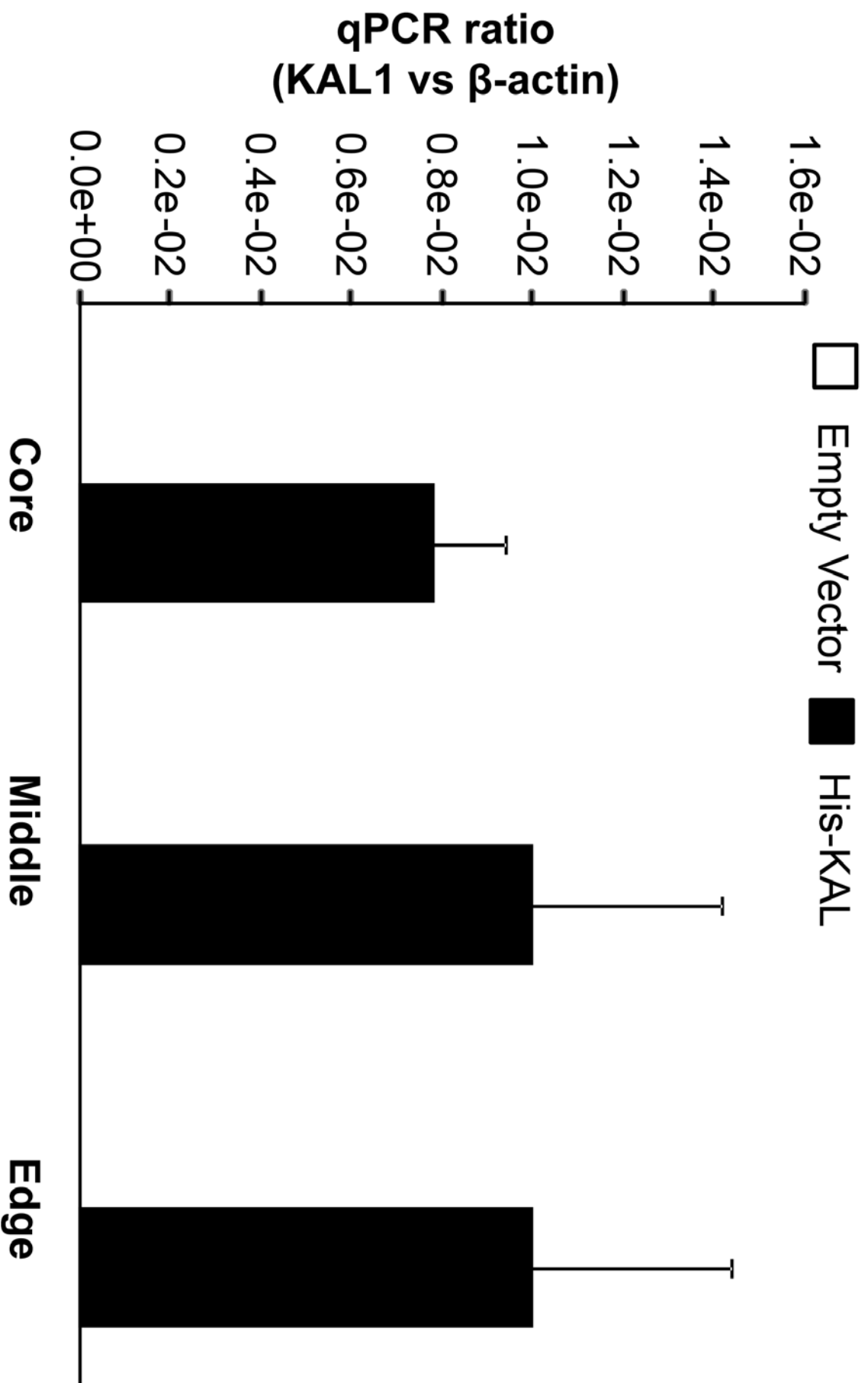

Supplement: Supplementary Data [file supp_ERC-13-0181_Supplementary_figure_3.pdf]
